# Development of Highly Efficient Lamb Wave Transducers toward Dual-Surface Simultaneous Atomization

**DOI:** 10.3390/s24175607

**Published:** 2024-08-29

**Authors:** Chenhui Gai, Qinghe Ma, Jia Ning, Yizhan Ding, Yulin Lei, Honggeng Li, Chunhua Guo, Hong Hu

**Affiliations:** 1 School of Mechanical Engineering and Automation, Harbin Institute of Technology, Shenzhen 518055, China; 2 School of Advanced Engineering, Great Bay University, Dongguan 523000, China; 3 CNNC Shenzhen Group Co., Ltd., Shenzhen 523000, China

**Keywords:** surface acoustic wave, Lamb wave transducers, h/λ, electromechanical coupling coefficient, microfluidic atomization

## Abstract

Highly efficient surface acoustic wave (SAW) transducers offer significant advantages for microfluidic atomization. Aiming at highly efficient atomization, we innovatively accomplish dual-surface simultaneous atomization by strategically positioning the liquid supply outside the IDT aperture edge. Initially, we optimize Lamb wave transducers and specifically investigate their performance based on the ratio of substrate thickness to acoustic wavelength. When this ratio h/λ is approximately 1.25, the electromechanical coupling coefficient of A_0_-mode Lamb waves can reach around 5.5% for 128° Y-X LiNbO_3_. We then study the mechanism of droplet atomization with the liquid supply positioned outside the IDT aperture edge. Experimental results demonstrate that optimized Lamb wave transducers exhibit clear dual-surface simultaneous atomization. These transducers provide equivalent amplitude acoustic wave vibrations on both surfaces, causing the liquid thin film to accumulate at the edges of the dual-surface and form a continuous mist.

## 1. Introduction

Surface acoustic wave (SAW)-based atomization represents a significant advancement in the field of microfluidic technology [1,2,3]. Its primary merits lie in its low power requirements and the ease of fabrication, making it a cost-effective solution. The non-invasive characteristics of SAW-based atomization extend its applicability to a wide range of fields. The ability of SAW-based atomizers to generate micrometer-sized droplets without the need for moving parts or nozzles underscores their efficiency and adaptability [4,5,6]. The miniaturization potential of this technology, transforming a complete laboratory into a chip, enhances its portability and user convenience. In the realm of drug delivery, the aerosol concentration and size distribution produced by SAW atomization aligns with the requirements for asthma treatment. Moreover, microfluidics uses nanopumps for liquid delivery and special channels, through which the liquid is delivered with the help of surface acoustic waves. The operation of SAW atomization at a higher frequency (>10 MHz) compared to conventional ultrasonic atomization (<1 MHz) facilitates the generation of a substantial amount of aerosol in the 1–10 μm range [7,8,9]. The efficiency, versatility, and broad applicability of SAW-based atomization underscore its value in various scientific and technological domains [9,10,11].

While promising, SAW-based atomization also faces challenges and areas for improvement, making it a dynamic field for innovation. The conventional experiments are conducted using Rayleigh mode surface acoustic waves, which concentrate the mechanical energy to propagate along the surface of the piezoelectric substrate with a ratio of substrate thickness to acoustic wavelength h/λ close to infinite [12,13,14]. However, the thickness is limited, in fact, and the ratio h/λ would be lower. When h/λ approaches 1, the mechanical energy along the surface could leak to the bottom surface of the piezoelectric plate, then converting to a Lamb wave [15,16,17]. Although Lamb waves also play an important role in microfluidics, they do not receive as much attention as Rayleigh waves. The reason for receiving less mention could be summarized as its extreme complexity, but it also attracts researchers to investigate [18,19,20]. HYDRA has been reported as the recombination with the SAWs and traveling leak waves when h/λ≈ 1, termed as a surface reflected bulk wave, can achieve an atomization rate of up to 8 mL/min [21,22,23]. A high-order Lamb acoustic wave could realize the vertical jetting array of droplets with h/λ≈ 1. However, it is still unclear how the dispersion effect of Lamb waves affects the driving of microfluidics, even atomization. The research of h/λ optimized selection is still insufficient, and the phenomenon of Lamb waves-driven droplet atomization has not been fully explored [13,24,25].

Here, we start with the simulation analysis from a piezoelectric substrate to a finite plate to carefully ascertain the dispersion effect and electromechanical coupling coefficient $K^{2}$ of 128° Y-X LiNbO_3_ and optimize the design of parameters to realize highly efficient Lamb wave transducers. Subsequently, we conduct experiments to test the reflection S_11_ spectra and verify the simulation results, then we investigate the Lamb waves propagation characteristics through the droplet transfer process. Finally, the mechanisms of liquid film movement and dual-surface simultaneous atomization are studied by positioning the liquid supplement paper strips outside the IDT aperture, at the edge of the piezoelectric plate, near the Lamb wave transducers.

## 2. Simulation and Design of SAW Transducers

### 2.1. Governing Equations

Let us consider a coupled acoustic wave equation in piezoelectric media (without external mechanical stress or displacement) [26]:

(1) $$\left\{\begin{array}{cc} c_{ijkl}\frac{\partial^{2}u_{k}}{\partial x_{l}\partial x_{j}}+e_{kij}\frac{\partial^{2}\Phi }{\partial x_{k}\partial x_{j}} & =\rho \frac{\partial^{2}u_{i}}{\partial t^{2}}\\ e_{ikl}\frac{\partial^{2}u_{k}}{\partial x_{l}\partial x_{i}}-\varepsilon_{ik}\frac{\partial^{2}\Phi }{\partial x_{k}\partial x_{i}} & =0 \end{array}\right.\;(i,j,k,l=1,2,3),$$

where ρ is the density of media, $u_{i}$ is the displacement along the $x_{i}$ direction, *t* is the time, Φ is the potential, and $c_{ijkl}$, $e_{kij}$, $e_{ikl}$, $\varepsilon_{ik}$ are elastic constants, piezoelectric stress constants, and dielectric permittivity, respectively. Obviously, Equation (1) is a system of second-order homogeneous partial differential equations. An accurate solution for acoustic waves in any piezoelectric medium is all based on this governing equation through a set of specific boundary conditions.

**Figure 1:**
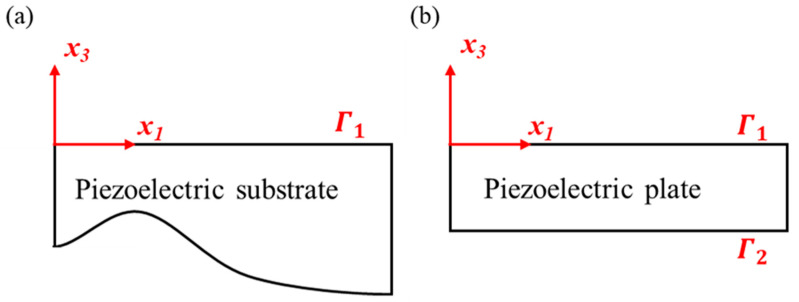
Coordinates for (**a**) piezoelectric substrate and (**b**) plate. $\Gamma_{1}$ and $\Gamma_{2}$ present the top surface and the bottom surface, respectively. The piezoelectric substrate is for Rayleigh waves, and it only has one boundary condition since the substrate is considered to have infinite thickness. In contrast, Lamb waves exist in the finite thickness piezoelectric plate with both top and bottom surface boundary conditions.

One can use Figure 1 to discuss specific boundary conditions of acoustic waves in piezoelectric media. Then, we can define that the normal stress component is zero at the surface of piezoelectric media as the mechanical boundary conditions:

(2) $$\begin{array}{c} \sigma_{i3}{|}_{\Gamma_{1}}=0\;\mathrm{for}\;\mathrm{Figure}1a\\ \sigma_{i3}{|}_{\Gamma_{1}}=\sigma_{i3}{|}_{\Gamma_{2}}=0\;\mathrm{for}\;\mathrm{Figure}1b, \end{array}$$

where $\sigma_{i3}$ represents the normal stress in three directions.

The electrical boundary conditions include a free surface or metalized surface. A metalized surface is considered to have a thin metal layer deposited on the propagation area of acoustic waves; it is also known as short-circuit. The free surface corresponds to a free-circuit or open-circuit. As the normal component electric displacement $D_{3}$ is discontinuous at the surface, the surface charge density γ is equal to the jump of $D_{3}$ across the surface, for example:

(3) $$D_{3}{|}_{\Gamma_{1}^{+}}-D_{3}{|}_{\Gamma_{1}^{-}}=\gamma .$$

It is known as a metalized surface, and the normal component of electric displacement is related to the surface potential Φ. $D_{3}{{|}_{\Gamma_{1}^{+}}=\eta \Phi D_{3}|}_{\Gamma_{1}^{-}}$, where $\eta =k_{1}\varepsilon_{0}$, $\varepsilon_{0}=8.845\times 10^{-12}$ is the vacuum permittivity.

Another one is the free surface. Due to the insulating properties of piezoelectric media, the surface charge density γ is equal to zero. It means that the electric potential is continuous, for example:

(4) $$D_{3}{|}_{\Gamma_{1}^{+}}-D_{3}{|}_{\Gamma_{1}^{-}}=0.$$

By associating governing equations and boundary conditions, we can accomplish the displacement fields of Rayleigh waves and Lamb waves. However, we can decompose the Lamb waves into asymmetric flexure-modes (A-Modes) and symmetric dilation-modes (S-Modes). The characteristic of the A-Modes is that the upper surface displacement is parallel to the lower surface displacement, while the characteristic of S-Modes is that the upper surface displacement is symmetrical to the lower surface displacement.

### 2.2. Electromechanical Coupling Coefficient

Electromechanical coupling coefficient $K^{2}$ demonstrates the ratio of stored mechanical energy to input electrical energy, or the ratio of stored electrical energy to input mechanical energy. For acoustic wave transducers, it can be thought as the strength of the excited acoustic wave modes. There is an Ingebrigtsen approximate expression [27]:

(5) $$K^{2}\approx \frac{2(v_{f}-v_{m})}{v_{f}}\times 100\%,$$

where $v_{f}$ is the phase velocity at the free surface and $v_{m}$ is that at the metalized surface.

The equation contributes a convenient way to calculate the electromechanical coupling coefficient whether in theoretical calculations or experimental tests.

### 2.3. COMSOL Simulation

**Figure 2:**
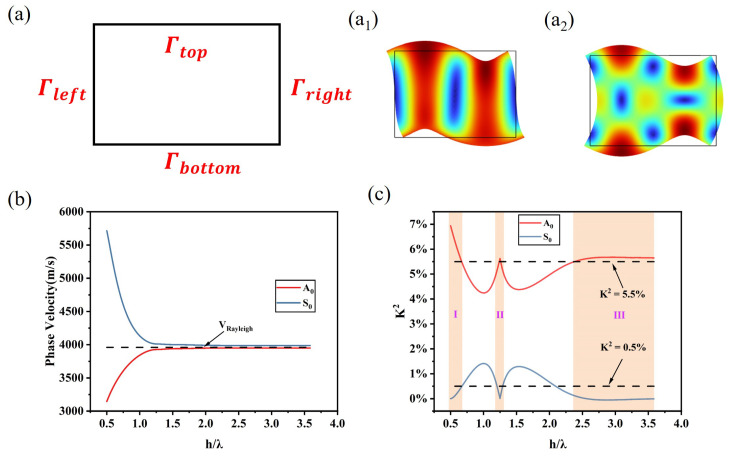
COMSOL simulation model and results. (**a**) Simplified two-dimensional piezoelectric plate model, (**a1**,**a2**) demonstrates the asymmetric flexure-mode and symmetric dilation-mode of lamb waves. (**b**) The phase velocity of the A_0_-mode and S_0_-mode varies with the ratio of thickness to wavelength. The dotted line represents the Rayleigh wave velocity; the velocity of the A_0_-mode and S_0_-mode are getting closer to that of the Rayleigh wave. (**c**) The electromechanical coupling coefficient $K^{2}$ varies with the ratio of thickness to wavelength. The upper dotted line manifests the nominal electromechanical coupling coefficient of 128° Y-X LiNbO_3_, 5.5%, which is also considered the electromechanical coupling coefficient of Rayleigh waves. The lower dotted line represents $K^{2}$ = 0.5%.

Here, one can use eigenfrequencies, analyzing in COMSOL software, to obtain $K^{2}$. First, we simplify the structure of a three-dimensional acoustic resonator to a two-dimensional one as shown in Figure 2a. $\Gamma_{top}$, $\Gamma_{bottom}$, $\Gamma_{left}$, and $\Gamma_{right}$ are the boundary conditions. The periodic structural parameters and boundary conditions are given in Table 1, in which the electrode width varies from 24 μm to 175 μm by using parameterized scanning. Next, we define the bottom surface as the free surface and change the top surface from the free surface to the metalized surface to gain the frequency of the free and metalized surface. Note that one should set the surface charge density γ=0 for free electrical boundary conditions, and Φ=0 for metalized electrical boundary conditions. Finally, we can extract the frequencies of zero-order asymmetric flexure-modes and symmetric dilation-modes as shown in Figure 2a1,a2. The Ingebrigtsen approximate expression can be rewritten as:

(6) $$K^{2}\approx \frac{2(v_{f}-v_{m})}{v_{f}}\times 100\%=\frac{2(f_{f}-f_{m})}{f_{f}}\times 100\%,$$

where $f_{f}$ and $f_{m}$ are frequencies of the free and metalized surface, respectively.

### 2.4. Design of SAW Transducers

Once we extract the frequencies of zero-order asymmetric flexure-modes and symmetric dilation-modes, the phase velocity of both modes is known through f=v/λ=v/(4×a). Then, Figure 2b demonstrates that the velocity of A_0_-mode increases as the ratio increases, and the S_0_-mode is opposite to the A_0_-mode. The velocity of both modes approaches the Rayleigh wave velocity when the piezoelectric plate trends to the piezoelectric substrate. As shown in Figure 2c, three regions are identified based on the value of the electromechanical coupling coefficient. In Region I, Region II, and Region III, the electromechanical coupling coefficient of the A_0_-mode is close to 5.5%, the nominal value of $128^{\circ }$ Y-X ${\mathrm{LiNbO}}_{3}$; we can assume that the efficiency of the stimulating acoustic waves is sufficiently high in these regions. In contrast, the electromechanical coupling coefficient of the S_0_-mode is lower than $K^{2}=$ 0.5% and approaching zero infinitely with the increase in the ratio of the thickness to wavelength. It means that S_0_-mode Lamb waves will not be excited in a piezoelectric semi-infinite substrate [28,29].

Note that Region II represents the ratio of thickness to wavelength being around 1.25, and the electromechanical coupling coefficient $K^{2}$ of the A_0_-mode is extremely close to that of Rayleigh waves. Meanwhile, that of S_0_-mode is approximate zero. As previously reported, when setting the ratio close to 1.25, the A_0_-mode waves may redevelop at the bottom surface. They define the waves as the reflected bulk wave [5,21], i.e., the SRBW, constructively recombined with the SAW that, in turn, leads to further strengthen the SRBW. Hence, the A_0_-mode at Region II may hold sufficient mechanical energy to maintain the meeting of leaky waves and A_0_-mode waves wrapping around the edge.

According to Region II in Figure 2c, we design the SAW transducers mainly depending on the ratio of thickness to wavelength. The thickness of $128^{\circ }$ Y-X ${\mathrm{LiNbO}}_{3}$ is 350 μm, and the wavelength is set as 132 μm, 280 μm, 320 μm, 352 μm, and 468 μm; thus, the ratio of thickness to wavelength is 2.65, 1.25, 1.09, 0.99, and 0.75. The uniform interdigital transducer is attempted to conduct experiments with the 3 mm IDT aperture and 30 pairs in total as shown in Table 2.

## 3. Experimental Section

### 3.1. Fabrication of SAW Transducers

The SAW transducers are fabricated via the standard lithography and liftoff technique. The wafer undergoes a cleaning process using acetone and methanol, followed by rinsing with deionized (DI) water. A uniform layer of photoresist (PR, AZ5214E, Clariant Company, Muttenz, Switzerland) is then spin-coated onto the wafer and subjected to an initial exposure. A 150 nm-thick aluminum film is deposited onto the patterned LiNbO_3_ wafer using an E-beam evaporator. The top pattern is revealed after immersing the wafer in acetone and performing a lift-off process. The gel (3M, VHB, Tape 4905, Hong Kong, China) is employed to absorb acoustic waves, where both its sides can bond to a broad range of high-surface-energy substrates including metals, glass, and easier-to-bond paints and plastics.

### 3.2. Experimental Setup

The frequency characteristics are measured via a network analyzer (E5073C, Agilent, Hong Kong, China). The RF electrical signal is generated by the signal generator (RIGOL DSG3030) and amplified by the power amplifier (Mini-circuit LZY-22). Acoustic waves could make droplets deform, transfer, and atomize. Though the process lasts for a short time, it could be recorded by a high-speed camera (CP70-1 HS-M-1900, Optronics, Germany) and super macro lens (LW-FF 25 mmf/2.8 ULTRA MACRO 2.5–5.0X, LAOWA, Hong Kong, China).

**Figure 3:**
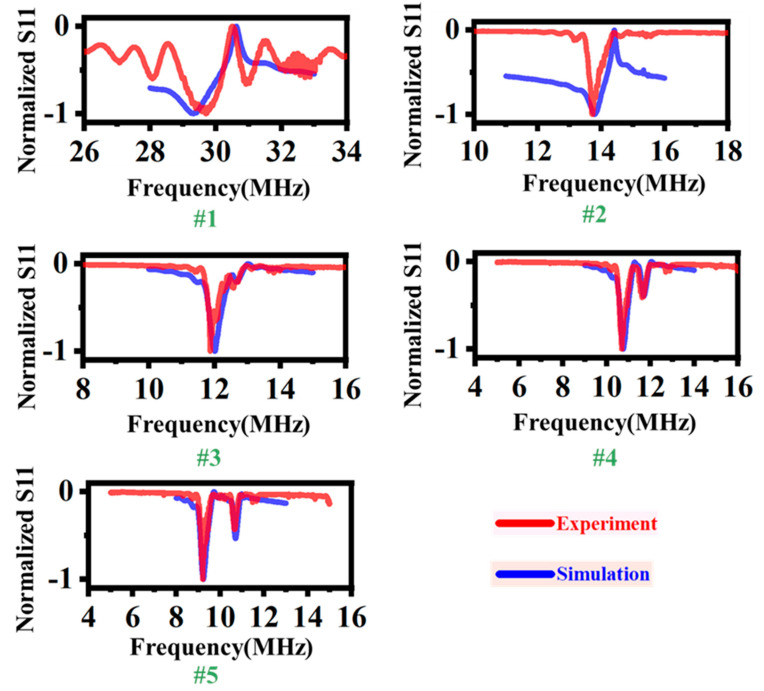
Reflection S_11_ spectra of SAW transducers with the designed wavelength of 132 μm, 280 μm, 320 μm, 352 μm and 468 μm. With the increasing wavelength, the transducers are named #1, #2, #3, #4, and #5. The experimental results (red lines) agree with the simulation results (blue lines).

We divide the SAW transducers into three types according to the ratio of thickness to wavelength, h/λ≈3 (Figure 3(#1)), h/λ≈1.25 (Figure 3(#2)), and h/λ≈0.75 (Figure 3(#5)), to explore the propagating direction of acoustic waves generated by these types transducers. The gel is fixed at the right side to absorb acoustic waves and prevent the acoustic waves from transferring from the top surface to the bottom surface after multiple reflections at the edge of the piezoelectric plate. A sessile droplet is placed on the top surface on the left side of IDT. Due to different types of acoustic waves that could cause different droplet transfer motions, we would further fix the gel on the bottom surface or left edge of the piezoelectric plate to further clarify the propagating direction of the acoustic waves.

In order to further demonstrate acoustic wave characteristics, there are methods to implement the visualizing acoustic waves in real-time mode, such as the X-ray topography method [30,31] and the Laser Doppler Vibrometer [32] method. For the Lamb wave transducers, we use a Laser Doppler Vibrometer (LDV, UHF-120, Polytec PI, Baden-Württemberg, Germany) to measure the displacement in the direction normal to the surface.

## 4. Results and Discussion

The S_11_ spectrum of SAW transducers is shown in Figure 3. We normalize the S_11_ value to give prominence to its frequency response. As a result, the fabrication process will lead to deviations between the experimental measurement and simulation of S_11_ values. Through f=v/λ, we find that the experimental results agree with the COMSOL simulation results in the frequency response, where *v* is the phase velocity of acoustic waves, which is clearly shown in Figure 2b. The #1 transducer in Figure 3 is considered as the typical Rayleigh wave transducer, although it does not propagate in an infinite thickness substrate. The ratio of the thickness to wavelength is around 2.65, and its phase velocity is very close to the Rayleigh waves. The rest of the transducers (#2–#5) are Lamb wave transducers, and #2 corresponds to Region II in Figure 2c. The #2 frequency shows only one minimal value that means that the A_0_-mode Lamb wave is 13.9 MHz, and the S_0_-mode Lamb wave has degenerated due to the electromechanical coupling coefficient being lower than 0.5%. The frequency of the A_0_-mode and S_0_-mode Lamb wave for the #3–#5 transducers can be distinguished clearly, and the difference in resonant frequency between the A and S modes is increasing, which conforms to the dispersion characteristics of Lamb waves.

**Figure 4:**
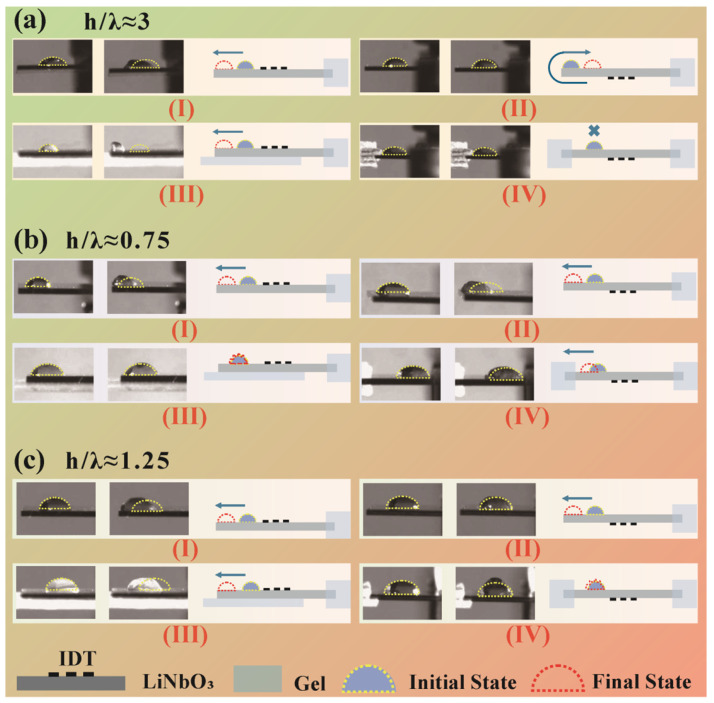
Droplet transfer or deformation, excited by a series of SAW transducers. (**a**) h/λ≈3, typical Rayleigh wave transducers, (**b**) h/λ≈0.75, wavelength larger Lamb waves, (**c**) h/λ≈1.25, thickness larger Lamb waves. The yellow dotted lines and blue semicircles indicate the initial positions of the droplet, the red dotted lines show the final positions of the droplet, and the light blue rectangles represent the acoustic absorbing gel.

A long-standing belief is that Lamb waves have an order of magnitude less driving efficiency for microfluidics than Rayleigh waves. The reason is that the acoustic energy is concentrated along the surface of the substrate for the Rayleigh waves, and the large strain could almost leak into the microfluidic flows at the solid–liquid interface. In our experiments, we will carefully explore the capability of microfluidics of Rayleigh waves and Lamb waves through a range of SAW transducers. Firstly, we can realize that there is an obvious contrast between the #2 transducer and #3–#5 transducers, although they are all Lamb waves. That is, for the #2 transducer, a complete acoustic wavelength vibration could travel through the entire plate thickness as a result of h/λ≈1.25, which is similar to the Rayleigh waves.Secondly, the #2 transducer achieves the same high electromechanical coupling coefficient compared to the #1 transducer; it is shown in Figure 2c. Lastly, the Lamb wave transducers would not give rise to a large temperature gradient across the surface region thickness; hence, it would not cause the transducers’ failure compared to Rayleigh wave transducers [33]. As shown in Figure 4, we chose three typical transducers to investigate the propagating direction of acoustic waves.

The conventional Rayleigh wave transducer is manifested in Figure 4a as its h/λ≈3. In Figure 4a(I), the Rayleigh waves push the droplet transfer to the left, which agrees with the direction of acoustic wave propagation. When the droplet is placed on the bottom surface as shown in Figure 4a(II), the droplet moves in the opposite direction. If we do not fix the gel on the left edge of the transducer, the acoustic waves will not be absorbed rather than having multiple reflections occur at the left edge of the piezoelectric plate, and then the acoustic waves will continue to propagate on the bottom surface. The droplet moves under the bottom surface of acoustic waves. Even if we fix the gel on the whole bottom surface (opposite to the surface deposited IDT), which could cover the IDT and droplet, Figure 4a(III), the droplet transfer is unaffected as a result of Rayleigh waves traveling along the surface. Instead of this setup, if we fix the gel on the left edge of the transducer, like Figure 4a(IV) shows, the droplet will not receive the effect of acoustic waves. It is easy to understand the phenomenon based on the Rayleigh wave propagation characteristics.

For Lamb waves, the situations are shown in Figure 4b,c. Here, we use antisymmetric Lamb waves to conduct experiments due to the high electromechanical coupling coefficient in Figure 2c and low return loss in Figure 3. When h/λ≈0.75, the droplet on the top surface can also move with the excited Lamb waves (Figure 4b(I)). The movement direction is the same as the case where the droplet is placed on the bottom surface (Figure 4b(II)); it is totally different from the Rayleigh waves because antisymmetric Lamb waves propagate along the same direction either on the top surface or the bottom surface. The Lamb waves do not need to wrap around the left edge to reach the other surface since they travel throughout the entire thickness. This is also the reason that the droplet just shows small deformation (Figure 4b(III)) under the same input power in Figure 4a(III). As demonstrated in Figure 4b(IV), when fixing the gel at the left-side edge of the piezoelectric plate, the droplet moves in the same direction as well.

As shown in Figure 4c, we continue to explore droplet movements when h/λ≈1.25. Although the cases are all Lamb waves in Figure 4b,c, there are some obvious differences in Figure 4c(III,IV). In Figure 4c(III), the droplet reveals a higher movement speed than that of Figure 4b(III). The key parameter h/λ may explain the difference; when h/λ≈0.75, the acoustic wavelength is smaller than the thickness, which means one whole acoustic wave has not completed its motion, just as the elliptical motion trajectory of particles has not been completed. In contrast, one completed acoustic wave has occurred, and the next wave continues with forward fluctuation when h/λ≈1.25. The completed waves may have a greater strengthening effect on the top surface, which explains the total opposite experimental phenomena well between two types of Lamb waves in Figure 4b,c.

**Figure 5:**
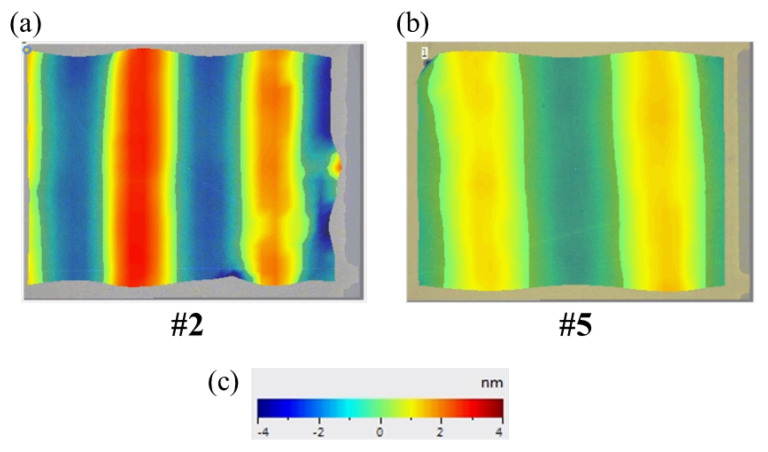
Lamb waves characterization using laser Doppler vibrometry (LDV) when the input power is 0.65 W. (**a**) The #2 Lamb wave transducers case (h/λ≈ 1.25), (**b**) the #5 Lamb wave transducers case (h/λ≈ 0.75), and (**c**) the amplitude corresponding to the scale bar from −4 nm to 4 nm. All LDV views denote a 800 μm length and a 600 μm width.

The characteristics of Lamb waves, especially for different h/λ, need to be investigated in detail. Figure 5 reveals the Lamb wave transducers for #2 and #5. The vibration amplitude scale bar is from −4 to 4 nm, and we can find that the displacement of #2 is obviously higher than that of #5. It means that #2 can provide stronger actuation capability.

**Figure 6:**
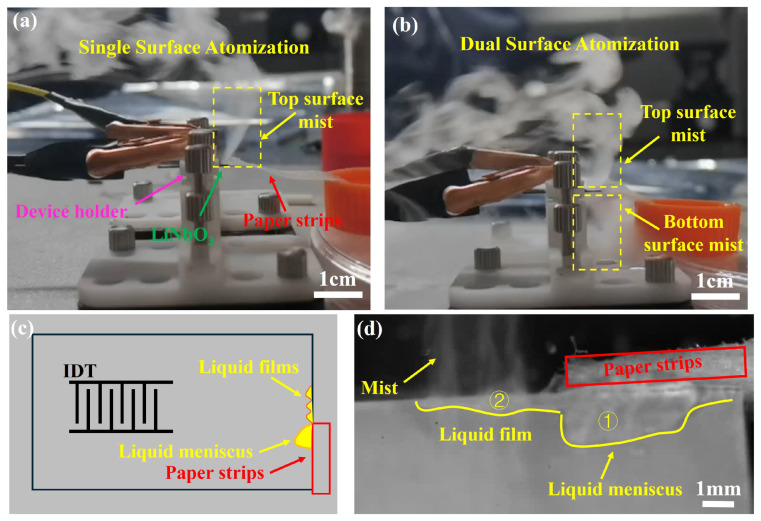
Schematic illustration of the SAW atomization process with liquid supplementation via paper strips. (**a**) Single surface atomization from the top surface using Rayleigh wave transducers (Figure 3(#1)), (**b**) dual-surface simultaneous atomization from both the top and bottom surfaces using Lamb wave transducers (Figure 3(#2)), (**c**) a schematic diagram illustrating the liquid movement and experimental setup during dual-surface simultaneous atomization driven by Lamb wave transducers, and (**d**) an enlarged view depicting the mechanism of the dual-surface simultaneous atomization process when driven by Lamb waves. The atomized liquid film (yellow region ➁) has not yet connected with the liquid meniscus (yellow region ➀) at the edge of the paper strip. The pink lines represent the transducer holder, the red lines denote the paper strips, the yellow indicators signify mist or liquid film, and the green lines indicate LiNbO_3_.

To investigate the acoustic wave excitation capable for microfluidics, we use Rayleigh wave transducers (Figure 3(#1)) and Lamb wave transducers (Figure 3(#2)) to atomize liquid with the input power at 3 W as shown in Figure 6a,b. The conventional Rayleigh waves atomization method is placing the paper strips on the surface of the transducer; the other side of the paper strips are connected to the deionized reservoir. Under the driving of Rayleigh waves, the thin liquid film is formed at the tip of the paper strip at the beginning, then atomization onset occurs when the film breaks up, and a plume of fine mist is ejected from the liquid film. Throughout this process, atomization just occurs on the top surface where Rayleigh waves propagate. However, we find from Figure 4d that the Lamb wave transducers (Figure 3(#2)) have the commensurate driving capability either on the top surface or the bottom surface, and we can take advantage of this propagation characteristic to atomize the liquid. As shown in Figure 6b, we connect the area outside the IDT aperture, at the edge of the piezoelectric plate, to the liquid supplement paper strip. Compared to Rayleigh waves atomization, the mist can be ejected from the dual surface of the top and the bottom. This phenomenon is consistent with the commensurate driving capability of the dual surface that we previously discovered.

For further understanding of the mechanism of dual-surface simultaneous atomization, we record the movement of the liquid film in Figure 6d. The bulk liquid meniscus is connected to the paper as usual (shown in the yellow region ➀). The liquid thin film is not in front of the bulk liquid meniscus as usual, instead appearing on the side close to the IDT aperture as illustrated in the yellow region ➁, and the dual-surface shows the same situation. This is a completely different phenomenon, and the necessary conditions for this phenomenon can be summarized as three categories: (1) the paper strips are contacting with the edge of the piezoelectric plate, (2) the contacting zone is the outside of the IDT aperture, and (3) the piezoelectric plate can provide commensurate amplitude acoustic wave vibration on the dual surface, which happens to be one of the characteristics of Lamb waves.

## 5. Conclusions

- We perceive that the electromechanical coupling coefficient $K^{2}$ of A_0_-mode Lamb waves can be as high as that of Rayleigh waves when the ratio h/λ≈1.25, and in this case, the S_0_-mode Lamb wave has degenerated due to its $K^{2}$ being lower than 0.5%.
- A_0_-mode Lamb waves propagation characteristics are not immutable with the change in h/λ. When h/λ≈0.75, the acoustic wavelength is smaller than the thickness, which means one whole acoustic wave has not completed its motion, and the bottom surface may hold a stronger driving ability. When h/λ≈1.25, the acoustic wavelength is larger than the thickness, and the completed acoustic waves may have a strengthening driving ability on the top surface.
- Compared to Rayleigh waves, Lamb waves can obtain a dual-surface simultaneous atomization phenomenon when putting the paper strips on the outside the IDT aperture, at the edge of the piezoelectric plate. This can be attributed to the fact that the piezoelectric plate can provide a commensurate amplitude acoustic wave vibration on the dual-surface. During the process of dual-surface simultaneous atomization, the liquid thin film is not in front of the bulk liquid meniscus as usual, instead appearing on the inside of the IDT aperture.

## Figures and Tables

**Table 1:** Settings of periodic structural parameters and boundary conditions.

	Electrode Width (a/μm)	Electrode Height (t/nm)	Plate Thickness ($h_{1}/\mu$m)
**Periodic Structural Parameters**	* 24:1:175	150	350
	$\Gamma_{\mathrm{left}}$, $\Gamma_{\mathrm{right}}$	$\Gamma_{\mathrm{top}}$	$\Gamma_{\mathrm{bottom}}$
**Mechanical Boundary Conditions** **Electrical Boundary Conditions **	Periodicity Periodicity	Free Free or metalized	Free Free

* Initial value: step size: terminal value.

**Table 2:** Design of SAW transducer.

Terms	Values
IDT pairs	30
IDT aperture	3 mm
Thickness	350 μm
Wavelength	132, 280, 320, 352, and 468 μm
h/λ	2.65, 1.25, 1.09, 0.99, and 0.75

## Data Availability

Data will be made available on request.

## Abbreviations

The following abbreviations are used in this manuscript:

SAW	Surface acoustic wave
IDT	Interdigital transducer
